# The Epidemiological Features and Pathogen Spectrum of Respiratory Tract Infections, Istanbul, Türkiye, from 2021 to 2023

**DOI:** 10.3390/diagnostics14111071

**Published:** 2024-05-22

**Authors:** Nuran Karabulut, Sema Alaçam, Esranur Şen, Mehmet Karabey, Nurhayat Yakut

**Affiliations:** 1 Departmant of Medical Virology, Basaksehir Cam and Sakura City Hospital, University of Health Science, 34480 Istanbul, Türkiye; 2 Departmant of Medical Microbiology, Istanbul Faculty of Medicine, Istanbul University, 34093 Istanbul, Türkiye; 3 Departmant of Pediatric Infectious Diseases, Bahçelievler Medipol Hospital, Istanbul Medipol University, 34196 Istanbul, Türkiye

**Keywords:** multiplex RT-PCR, SARS-CoV-2, RSV, influenzae viruses

## Abstract

Respiratory tract infections (RTIs) can lead to both recurrent seasonal epidemic outbreaks and devastating pandemics. The aim of this study was to evaluate the epidemiologic characteristics and pathogen spectrum of RTIs using a multiplex RT-PCR panel. A total of 9354 cases with suspected RTIs between February 2021 and July 2023 were included in this study. A total of 11,048 nasopharyngeal and oropharyngeal samples from these patients were analyzed for 23 respiratory tract pathogens using multiplex RT-PCR. *H. influenzae* and *S. pneumoniae* were considered as colonizing bacteria. At least one pathogen was detected in 70.66% of the samples; viral pathogens were detected in 48.41% of the samples, bacterial pathogens were detected in 16.06% of the samples, and viral + bacterial pathogens were detected in 35.53% of the samples. The most frequently detected viral pathogen was rhinovirus/enterovirus (RV/EV) (19.99%). Interestingly, in 2021, respiratory syncytial virus A/B showed atypical activity and replaced RV/EV as the most prevalent pathogen. Human bocavirus, *H. influenzae*, and *S. pneumoniae* were detected at higher rates in males (*p*: 0.038, *p*: 0.042, and *p*: 0.035, respectively), while SARS-CoV-2 and *B. pertussis* were detected at higher rates in females (*p* < 0.001 and *p*: 0.033). RTIs were found at higher rates in children (*p* < 0.001). SARS-CoV-2 and human coronaviruses 229E were detected at higher rates in adults (*p* < 0.001 and *p*: 0.001). This comprehensive study with a large sample size investigating RTI pathogens was the first in Türkiye. Understanding the current viral circulation using multiplex RT-PCR panels enables clinicians to predict the most likely pathogens affecting patients and contributes to patient management, in addition to anticipating potential threats.

## 1. Introduction

Respiratory tract infections (RTIs) are important causes of morbidity and mortality and affect public health worldwide. According to the World Health Organization (WHO), lower RTIs ranked as the fourth-highest global cause of death in 2019 [1]. Upper RTIs, which are among the most common diseases, are generally short-term, mild, and self-limiting infections, but they can cause serious complications such as pneumonia and myocarditis and impose a heavy burden on individuals and society [2].

Coronavirus disease 2019 (COVID-19), which emerged in Wuhan, China, at the end of 2019, caused a global pandemic. To date, over 774 million people have been infected and over 7 million have died worldwide [3]. With this latest pandemic, the potential threat of respiratory viruses has been recognized once again, and the etiological surveillance of respiratory infections has become even more important. Various measures have been implemented worldwide to prevent the transmission of severe acute respiratory syndrome coronavirus 2 (SARS-CoV-2) and control the outbreak [4]. These measures have resulted in not only a reduction in SARS-CoV-2 cases but also a reduction in other respiratory infections and changes in their epidemiology.

Nowadays, the diagnosis of RTI pathogens has improved significantly with the widespread use of real-time polymerase chain reaction (RT-PCR) diagnostic methods that can detect multiple pathogens. Investigating the epidemiology of RTIs is also critical for clinical diagnosis, treatment, and prognosis [5]. In this study, we determined the detection rates of RTI pathogens using a multiplex RT-PCR test platform in patients admitted with RTIs between 2021 and 2023. The epidemiologic characteristics of the RTIs were also evaluated, including sex, age, and seasonal variations of different pathogens. The novelty of this study lies in its comprehensive investigation of RTI pathogens utilizing a large sample size, marking the first such endeavor in Türkiye.

## 2. Materials and Methods

### 2.1. Study Design

A total of 9354 cases with suspected RTIs between February 2021 and July 2023 were included in this study. Most patients presented with mild and self-limiting respiratory and constitutional signs and symptoms, such as sneezing, nasal congestion and discharge, sore throat, cough, fever, headache, and malaise. Some of the patients were hospitalized, and some were treated as outpatients. The samples obtained from these patients were studied using multiplex RT-PCR tests at the University of Health Sciences, Basaksehir Cam and Sakura City Hospital, Medical Virology Laboratory. This hospital is the largest in the Marmara area of Türkiye and has 2682 beds. The study was approved by the ethics committee of Basaksehir Cam and Sakura City Hospital (protocol code: 2023/483-11.10.2023).

### 2.2. Specimen Collection

An oropharyngeal sample was first collected from patients with a swap, and then a nasopharyngeal sample was collected with the same swap and put into viral nucleic acid isolation tubes (vNAT, Bio-speedy, Bioeksen, Istanbul, Türkiye). These samples were transferred to the laboratory under cold chain conditions, stored in a refrigerator at 2–8 °C and analyzed within 24 h.

### 2.3. Nucleic-Acid-Based Pathogen Detection

Nucleic acid extraction of the samples was performed using the total nucleic acid extraction kit (Bioeksen, Istanbul, Türkiye), which is based on a magnetic beads method, on the Zybio EXM 3000 instrument (Zybio, Shenzhen, China) according to the manufacturer’s instructions.

After the nucleic acid extraction, respiratory pathogens were analyzed using the Bio-Speedy Respiratory RT-qPCR MX-24S panel kit (Bioeksen, Istanbul, Türkiye) on the Bio-Rad CFX96 Touch instrument (Hercules, CA, USA). The positive, negative, and internal controls (RNase P gene) were included in each run. The results of the tests were evaluated according to the manufacturer’s recommendations. The respiratory panel kit simultaneously detects the following 23 respiratory pathogens: SARS-CoV-2, influenza A virus (InfA), influenza B virus (InfB), human coronaviruses (CoV) 229E, 0C43, NL63, and HKU1, parainfluenza virus (PIV) 1, 2, 3, and 4, adenovirus, respiratory syncytial virus A/B (RSV), human metapneumovirus (MPV), human bocavirus (BoV), rhinovirus/enterovirus (RV/EV), parechovirus, *Legionella pneumophilia*, *Mycoplasma pneumoniae*, *Chlamydophila pneumoniae*, *Bordetella pertussis*, *Haemophilus influenzae*, and *Streptococcus pneumoniae*. *H. influenzae* and *S. pneumoniae* were considered as colonizing bacteria.

From the samples in which InfA was detected, the InfA subtypes were analyzed using the Bio-Speedy Influenza A/B/AH1-3-5-7-qPCR Panel kit (Bioeksen, Istanbul, Türkiye) on the Bio-Rad CFX96 Touch instrument (Hercules, CA, USA). The positive, negative, and internal controls (RNase P gene) were included in each run. The results of the tests were evaluated according to the manufacturer’s recommendations. The kit detects InfA-H1, InfA-H3, InfA-H5, and InfA-H7 subtypes.

### 2.4. Statistical Analysis

Statistical analyses were performed using the SPSS Statistics v21 program (SPSS Inc., Chicago, IL, USA). Visual (histograms) and analytical (Kolmogorov–Smirnov/Shapiro–Wilk’s test) methods were used to test the normality of the distributions of continuous variables. The Student *t*-test or Mann–Whitney U test was used to compare the parameters between the groups. The chi-square test or Fisher exact test was used for categorical comparisons of nominal values in different groups. A *p*-value of <0.05 was accepted as significant.

## 3. Results

### 3.1. Study Population

A total of 11,048 samples obtained from 9354 patients were included in this study. The age of the patients ranged from 0 to 98, with a median age of 4 years (interquartile range (IQR): 0–25), and 55.73% were male.

### 3.2. Overall Detection of Respiratory Tract Pathogens

One or more pathogens were detected in 7807 samples (70.66%); these were viral pathogens in 3779 (48.41%) samples, bacterial pathogens in 1254 (16.06%) samples, and viral + bacterial pathogens in 2774 (35.53%) samples.

**Figure 1:**
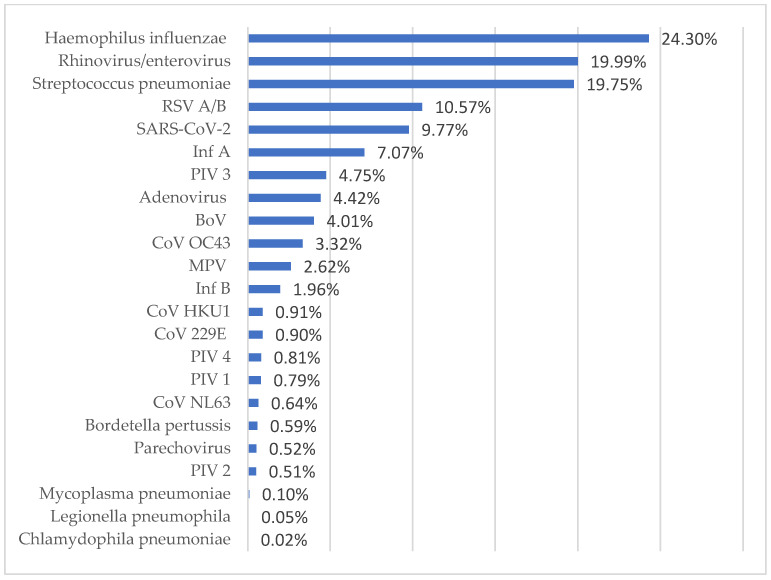
Detection rates of respiratory tract pathogens.

**Figure 2:**
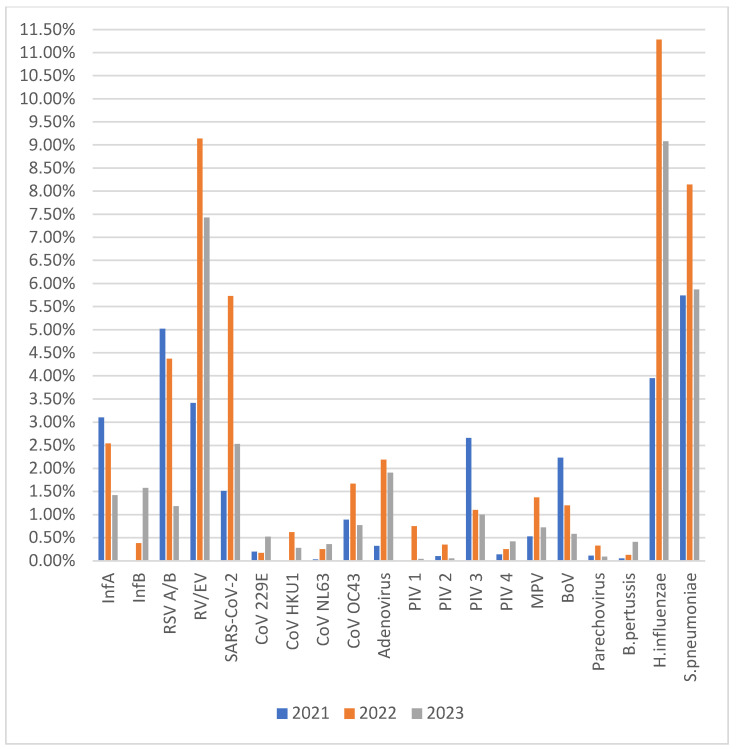
Detection rates of respiratory tract pathogens according to year.

The four most frequently detected viral pathogens were RV/EV, RSV A/B, SARS-CoV-2, and InfA; the most frequently detected bacterial pathogens were *H. influenzae* and *S. pneumoniae* (Figure 1). The most common viral pathogen in 2021 was RSV, and InfB was absent that year (Figure 2). The most common bacterial pathogen in 2021 was *S. pneumonia*. In both 2022 and 2023, the most common viral and bacterial pathogens were RV/EV and *H. influenza* (Figure 2).

A total of 254 InfA viruses were subtyped in 2022 and 2023; 50.79% (129/254) were H1 and 49.21% (125/254) were H3. In 2022, the dominant InfA subtype was H3 (63.04%, 87/138), while in 2023, it was H1 (67.24%, 78/116) (*p* < 0.001). Among the coronaviruses, SARS-CoV-2 (9.77%) was the most frequently detected, CoV OC43 (3.32%) was the second most frequently detected, and each of the other CoVs was detected at a rate below 1% (*p* < 0.001). PIV 3 (4.75%) was the most common parainfluenza virus, with each of the other PIVs detected at a rate below 1% (*p* < 0.001).

### 3.3. Co-Detection of Pathogens

**Figure 3:**
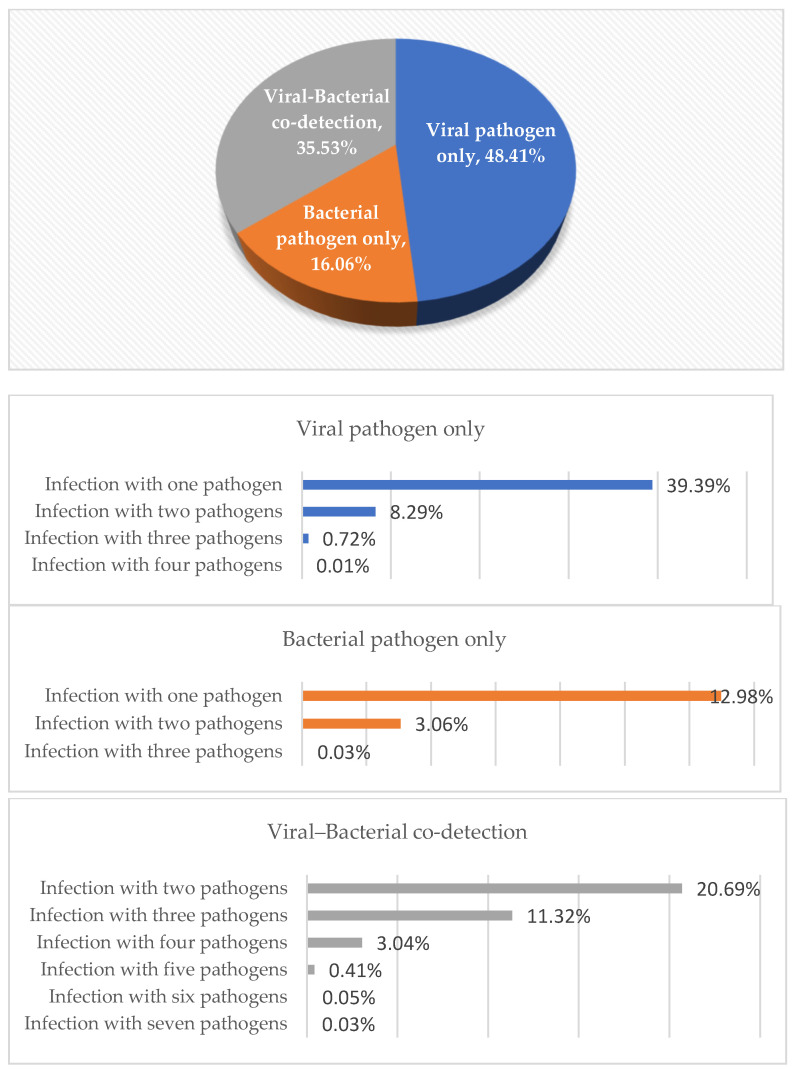
Single and multiple detection rates of the pathogens.

Viruses were the most frequently detected pathogens in the respiratory tract samples. The rate of detection of viruses alone in the respiratory tract samples ranked first with 48.41%. The rate of detection of viruses and bacteria together ranked second, with 35.53%, while the rate of detection of bacteria alone was 16.06% (Figure 3).

In the samples in which only viruses were detected, a single viral pathogen was detected in the majority (39.39%), while dual viral pathogens were detected in 8.29% and, rarely, triple or quadruple viral pathogens were co-detected. In the samples in which only bacteria were detected, a single bacterial pathogen was detected in the majority (12.98%), while dual bacterial pathogens were detected in 3.06% and, rarely, triple bacterial pathogens were co-detected. In the samples in which bacterial and viral agents were co-detected, the majority (20.69%) had two pathogens, 11.32% had three pathogens, 3.04% had four pathogens, and, rarely, five, six, or seven pathogens were co-detected (Figure 3).

**Figure 4:**
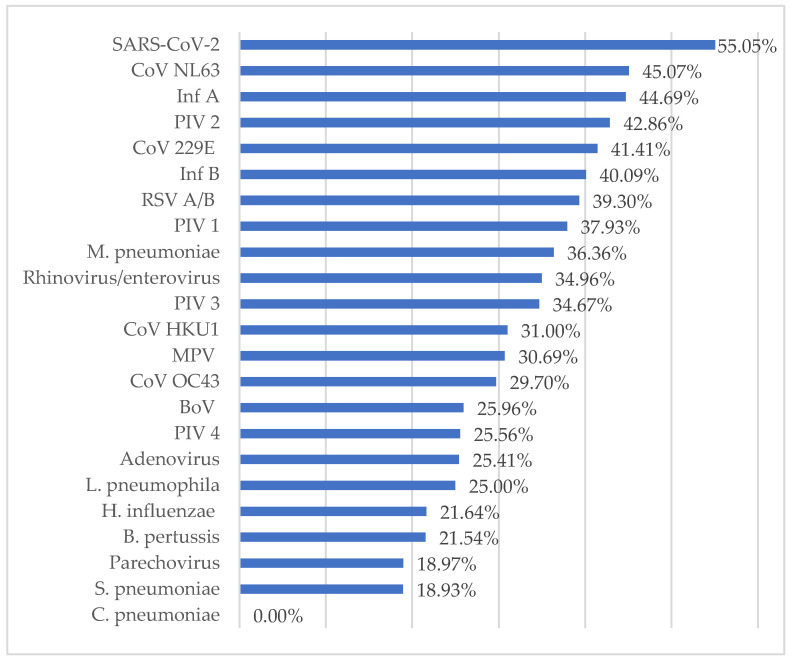
Single detection rates of the pathogens.

A single pathogen was detected in 4088 samples (52.36%), while more than one pathogen was detected in 3719 samples (47.64%). The single detection rates of pathogens from the respiratory tract samples are shown in Figure 4. The most frequently detected single pathogens were SARS-CoV-2, CoV NL63, and InfA, respectively. The most common dual pathogens were RV/EV + *H. influenza* at 12.44%, *H. influenza* + *S. pneumoniae* at 9.00%, and RV/EV + *S. pneumoniae* at 8% (Table 1).

### 3.4. Sex Distribution

**Figure 5:**
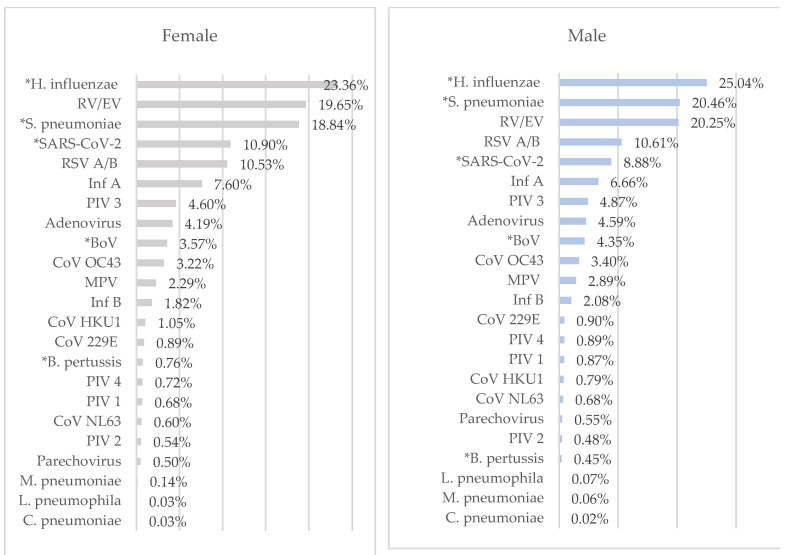
Distribution of respiratory tract pathogens by sex. * There is a statistically significant difference.

Of the 7807 patients with RTIs, 56.10% were male. No statistical difference was found between male and female patients in terms of RTIs (*p*: 0.889). The most frequently detected pathogens in females were *H. influenzae*, RV/EV, *S. pneumoniae*, SARS-CoV-2, RSV A/B, and InfA, respectively, while in males, they were *H. influenzae*, *S. pneumoniae*, RV/EV, RSV A/B, SARS-CoV-2, and InfA (Figure 5).

BoV, *H. influenza*, and *S. pneumoniae* had higher rates in males (*p*: 0.038, *p*: 0.042, and *p*: 0.035, respectively). However, SARS-CoV-2 and *B. pertussis* had statistically significantly higher rates in females (*p* < 0.001 and *p*: 0.033). There was no statistically significant difference between genders in terms of the detection rates of other respiratory tract pathogens (Figure 5).

### 3.5. Age Distribution

**Figure 6:**
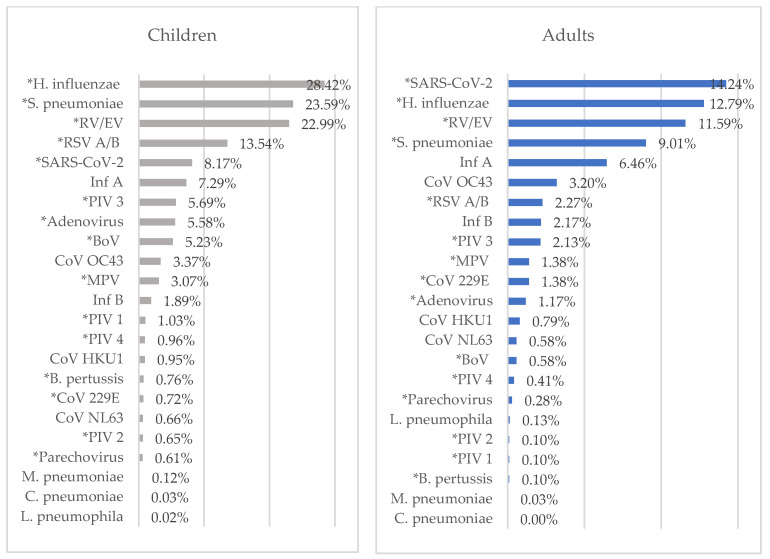
Distribution of respiratory tract pathogens in children and adults. * There is a statistically significant difference.

The median age of 7807 RTI cases with at least one pathogen was 3 years (IQR: 0–12). RTIs were statistically higher in children, at 77.29%, than in adults, at 52.13% (*p* < 0.001). In children, *H. influenzae*, *S. pneumoniae*, RV/EV, and RSV A/B were most frequently detected, while in adults, SARS-CoV-2, *H. influenzae*, RV/EV, and *S. pneumoniae* were most frequently detected, respectively (Figure 6). RV/EV (*p* < 0.001), RSV A/B (*p* < 0.001), MPV (*p* < 0.001), adenovirus (*p* < 0.001), PIV 1 (*p* < 0.001), PIV 2 (*p* < 0.001), PIV 3 (*p* < 0.001), PIV 4 (*p*: 0.005), BoV (*p* < 0.001), parechovirus (*p*: 0.003), *H. influenzae* (*p* < 0.001), *S. pneumoniae* (*p* < 0.001), and *B. pertussis* (*p* < 0.001) were detected at statistically higher rates in children. However, SARS-CoV-2 and Cor 229E were detected at statistically higher rates in adults (*p* < 0.001 and *p*: 0.001).

RTIs were found in 82.70% of individuals in the <1 year age group, 78.14% of those in the 1–2 years age group, 80.00% of those in the 3–6 years age group, 63.13% of those in the 7–17 years age group, 61.20% of those in the 18–35 years age group, 49.38% of those in the 36–60 years age group, and 47.90% of those in the >61 years age group.

**Figure 7:**
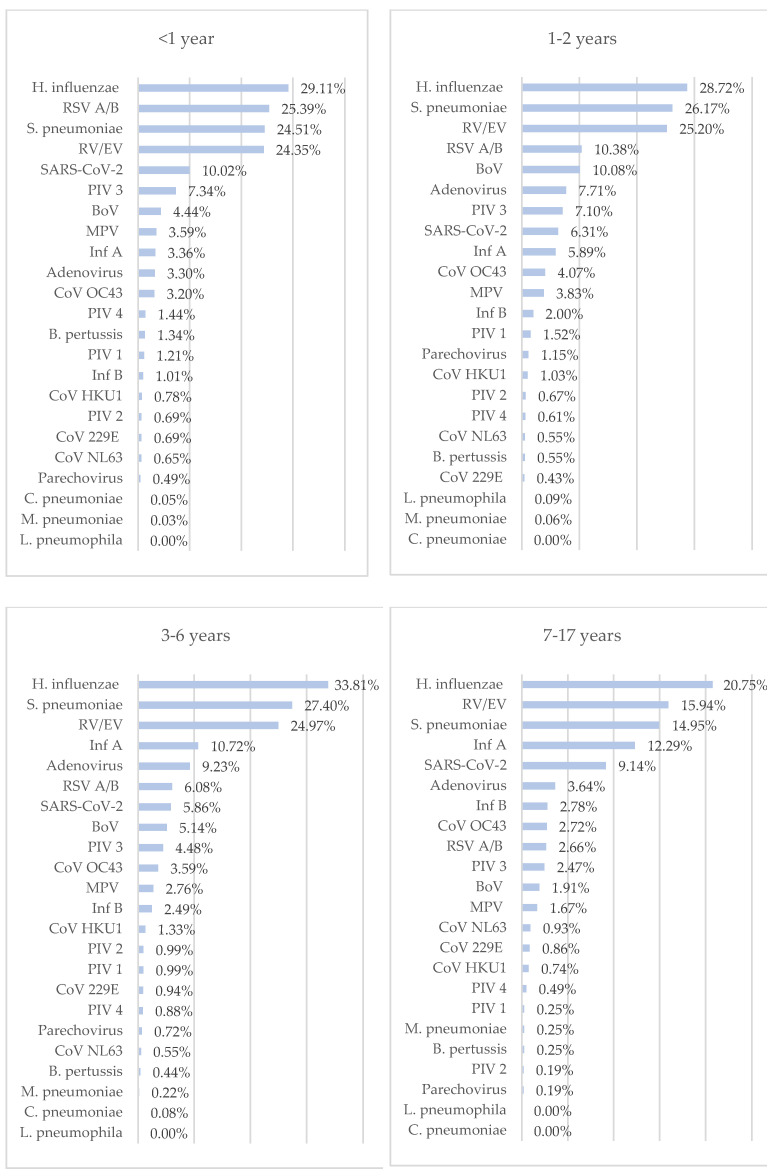

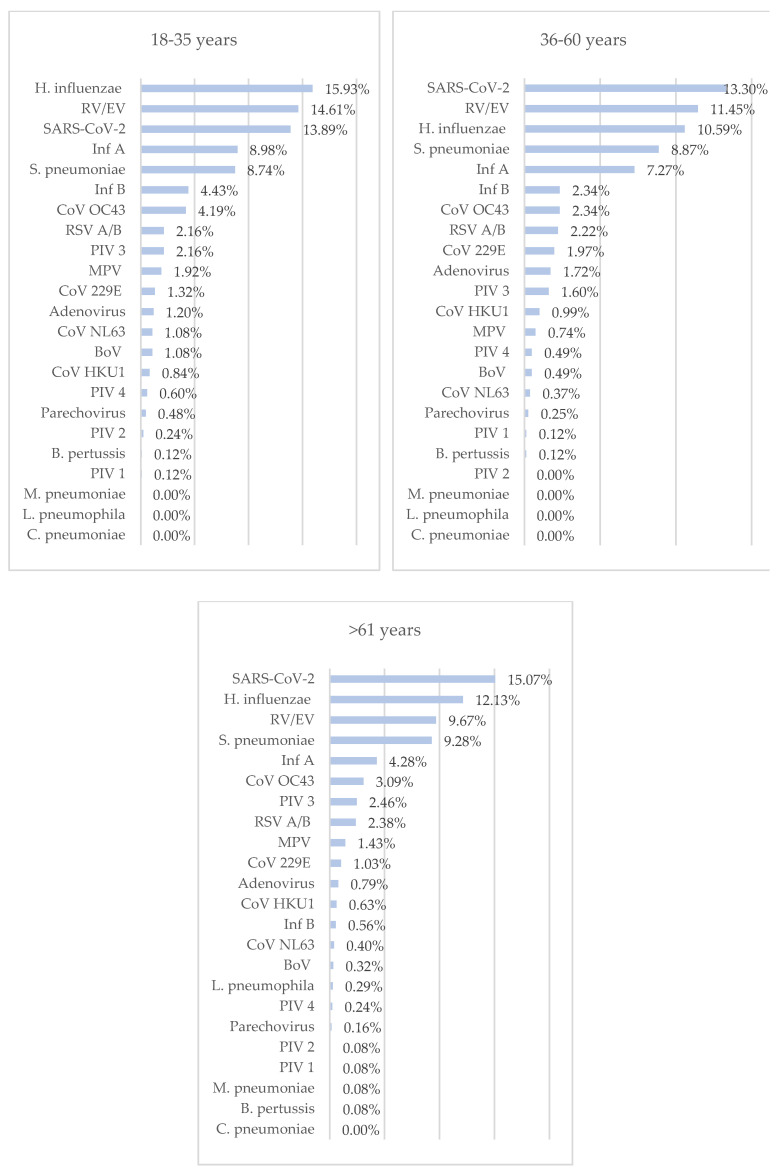
Distribution of respiratory tract pathogens by age group.

*H. influenza* was the most frequently detected pathogen in all groups except the 36–60 years and over 61 years age groups. RSV was the most frequently detected viral pathogen in the under 1 year age group, and RV/EV was the most frequently detected viral pathogen in the 1–2 years, 3–6 years, 7–17 years, and 18–35 years age groups. SARS-CoV-2 was the most frequently detected viral pathogen in the 36–60 and >61 years age groups (Figure 7).

### 3.6. Seasonal Distribution

**Figure 8:**
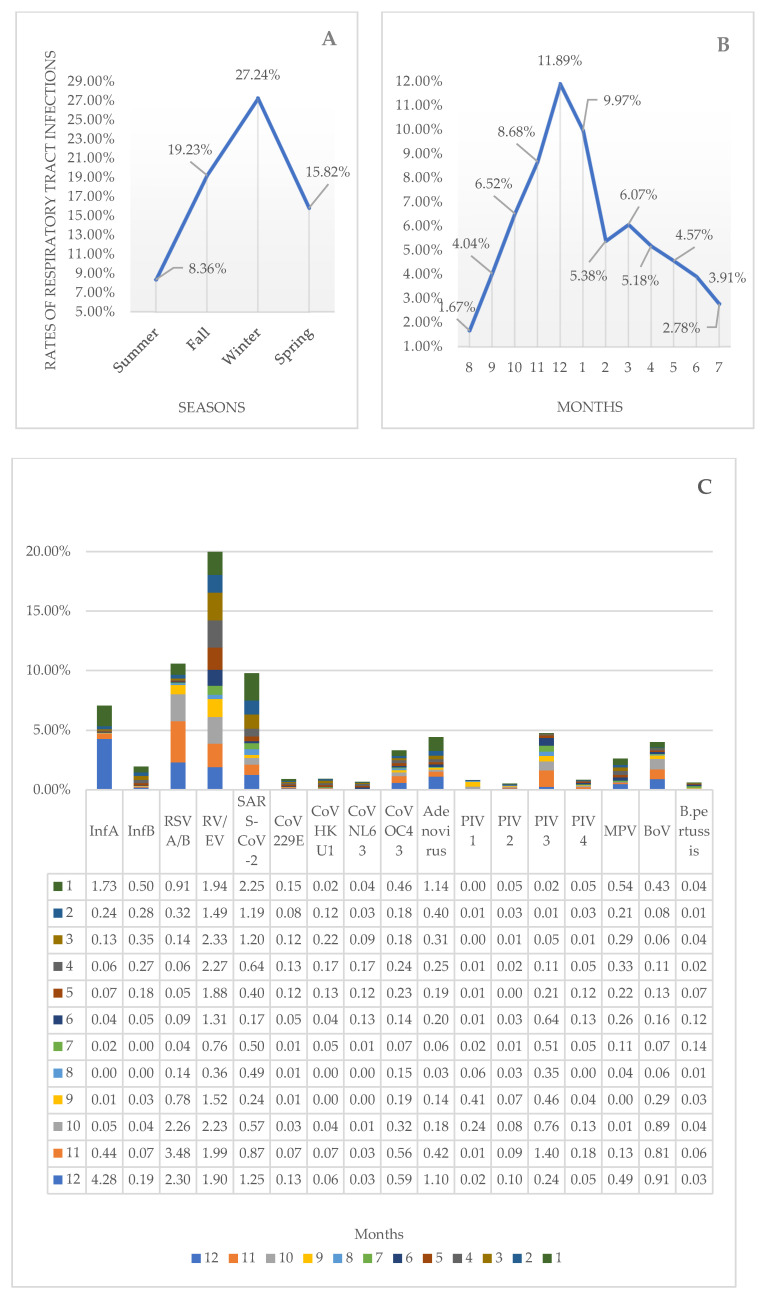
Rates of respiratory tract infections according to seasons (**A**) and months (**B**). Distribution of respiratory tract pathogens according to months (**C**).

RTIs were the highest in winter and the lowest in summer. The RTIs peaked in December, but dropped significantly in August. As shown in Figure 8, the most frequently detected pathogens were SARS-CoV-2, Cor 229E, adenovirus, and MPV in January; RV/EV and Cor NL63 in April; Cor HKU1 in May; *B. pertussis* in July; PIV 1 in October; RSV, PIV3, and PIV 4 in November; and InfA, InfB, BoV, Cor OC43, PIV 2, *H. influenza*, and *S. pneumoniae* in December.

## 4. Discussion

RTIs remain a significant economic and social burden on society. These infections are frequently caused by viruses, but bacteria are also responsible for a significant proportion of cases. With more than 200 known virus species circulating and the high mass incidence of upper RTIs, they have the potential to pose an unexpected threat to human health [2,6]. The multiplex RT-PCR assay, now a routine diagnostic test, is a good tool to understand current pathogen circulation and predict potential threats. The major strengths of this study were the large sample size and the use of a multiplex RT-PCR panel with a broad pathogen spectrum. In this study, at least one pathogen was detected in 70.66% of a total of 11,048 samples, and the majority of these cases were children. The main reason for the high positivity rate is that this test was performed mostly in patients with respiratory symptoms. In addition, bacterial colonization contributed to this high positivity rate.

In this study, the most frequently detected viral pathogen was RV/EV. RSV A/B, SARS-CoV-2, and InfA were other frequently detected viruses. Consistent with this study, many studies have also reported that rhinovirus is the most common respiratory pathogen [6,7,8,9,10]. Unlike most pathogens that cause respiratory infections, RV/EV is non-enveloped and is, therefore, resistant to environmental conditions and disinfectants, and can remain on surfaces for a long time. These characteristics contribute to RV/EV being the most common respiratory tract pathogen [6]. Besides the common cold, rhinovirus is associated with exacerbations of chronic obstructive pulmonary disease, asthma, and severe bronchiolitis in infants and children, as well as severe pneumonia in older people and some immunocompromised individuals [5].

Interestingly, our study revealed that in 2021, RSV showed atypical activity and replaced RV/EV as the most prevalent pathogen. In addition, InfB was not present in the same year. However, RV/EV remained the most prevalent viral pathogen in both 2022 and 2023. Similarly, a resurgence of RSV was reported in many regions of the world in 2021 [11,12,13,14]. In the first year of the COVID-19 pandemic, RSV cases, along with those of other viruses, declined rapidly worldwide due to the drastic measures taken to prevent transmission of pathogens [15]. The high activity of RSV in 2021 can be explained by reduced RSV immunity at the population level due to its long absence from circulation.

Viruses were the most frequently detected pathogens in the respiratory tract samples. The rate of detection of viruses alone in the respiratory tract samples ranked first, at 48.41%. The rate of detection of viruses and bacteria together ranked second, at 35.53%, while the rate of detection of bacteria alone was 16.06%. In the samples where only viruses or bacteria were detected, a single agent was detected in the majority of the samples. It has been suggested that infection with one viral pathogen prevents simultaneous infection by another virus through factors such as competition for resources, immune response, or viral proteins [16]. This mechanism may explain the current results showing a higher viral–bacterial but lower viral–viral co-infection rate. The most frequently detected single pathogens were SARS-CoV-2, CoV NL63, and InfA, respectively. In the majority of the samples where bacterial and viral agents were detected together, dual pathogens were detected. The most common dual pathogens were RV/EV + *H. influenza*, *H. influenza + S. pneumoniae*, and RV/EV + *S. pneumoniae*. Since the samples were taken from the upper respiratory tract, *H. influenza* and *S. pneumoniae* were considered as colonizing bacteria. The effects of bacterial colonization on current infection and its future effects could not be evaluated in this study. The clinical effects of bacterial colonization association with RTIs and the possible association between bacterial colonization and the occurrence of RTIs in the future should be investigated.

Consistent with WHO FluNet influenza reports [17], this study also revealed that the predominant InfA subtype was H3 in 2022 and H1 in 2023. While SARS-CoV-2 was detected most frequently among coronaviruses, CoV OC43 was the second most frequently detected. PIV 3 was the most common parainfluenza virus. It has also been shown in many studies conducted in different parts of the world that Cor OC43 among the four seasonal coronaviruses and PIV 3 among parainfluenza viruses were more common in circulation [18,19,20].

Similar to a previous study [6], no difference was found between the sexes in terms of RTIs in this study. However, in our study, BoV, *H. influenzae*, and *S. pneumoniae* were detected at higher rates in males, while SARS-CoV-2 and *B. pertussis* were detected at higher rates in females. Similarly, some studies have found BoV, *H. influenzae*, and *S. pneumoniae* more frequently in males [21,22,23]. Although SARS-CoV-2 has been associated with male sex in many studies, it was detected more frequently in females in this study, which is inconsistent with previous studies [24]. This inconsistency may be due to the fact that our laboratory mostly uses monoplex SARS-CoV-2 PCR instead of the respiratory multiplex RT-PCR panel in patients with suspected COVID-19. In addition to studies that did not find a relationship between the incidence of *B. pertussis* and sex, there are also studies showing that *B. pertussis* is detected more frequently in females [25,26].

RTIs have a higher incidence in children [6], and this was also the case in this study. Similar to previous studies [8,9,27], RV/EV and RSV A/B were most frequently detected in children, while SARS-CoV-2 and RV/EV were most frequently detected in adults. RV/EV, RSV A/B, MPV, adenovirus, PIV 1, PIV 2, PIV 3, PIV 4, BoV, parechovirus, *H. influenzae*, *S. pneumoniae*, and *B. pertussis* were detected at a statistically higher rate in children. However, SARS-CoV-2 and Cor 229E were detected at a statistically higher rate in adults.

RTIs were the highest in the <1 year age group and the lowest in the >61 years age group. The most frequently detected viral pathogen in the group under 1 year of age was RSV, and in the 1–2 years, 3–6 years, 7–17 years, and 18–35 years age groups, it was RV/EV. SARS-CoV-2 was the most frequently detected viral pathogen in the 36–60 and >61 years age groups. Many studies have reported that the highest incidence of upper RTIs was in children under five years of age [2]. In one study, consistent with our study, the highest rates of RTIs were reported in the infant and toddler groups, and the three most common pathogens were RSV, RV/EV, and *Mycoplasma pneumonia*. In addition, the lowest RTI rates were found in the young, middle-aged, and elderly groups [6].

RTIs were the highest in winter and the lowest in summer in this study, peaking in December but dropping significantly in August. The most frequently detected pathogens were SARS-CoV-2, Cor 229E, adenovirus, and MPV in January; RV/EV and Cor NL63 in April; Cor HKU1 in May; *B. pertussis* in July; PIV 1 in October; RSV, PIV3, and PIV 4 in November; and InfA, InfB, BoV, Cor OC43, PIV 2, *H. influenzae*, and *S. pneumoniae* in December. Changes in the host’s immune response, nutrition, and behavioral patterns due to changing weather conditions or climate changes are thought to influence the seasonal distribution of respiratory tract pathogens [28]. The peak of infection usually occurs during winter in temperate regions [8,9,28].

Due to the high cost of multiplex RT-PCR testing, monoplex SARS-CoV-2 PCR testing is performed separately for patients with suspected COVID-19 in our hospital. Therefore, SARS-CoV-2 rates may have been affected in this study using a multiplex RT-PCR panel. Since the study was retrospective, the clinical implications of bacterial colonization or co-detection pathogens could not be investigated. In recent years, there have been significant advances in molecular diagnostic tests, resulting in increased diagnostic success. Therefore, studies using new diagnostic methods have affected the pathogen spectrum [6]. In addition, it becomes difficult to compare the results between studies due to different diagnostic techniques and sample diversity.

## 5. Conclusions

Analyzing the detection rate of respiratory tract pathogens using multiplex RT-PCR panels is important for public health, clinical diagnosis, and treatment, as well as for the collection of epidemiological data. In addition, the approach can make important contributions to developing targeted preventive measures, patient management and treatment, and preventing unnecessary antibiotic use.

## Figures and Tables

**Table 1:** Distribution of dual pathogens.

Pathogens	N	%	Pathogens	N	%
RV/EV + *H. İnfluenzae*	311	12.44	PIV 4 + RV/EV	8	0.32
*H. İnfluenzae + S. pneumoniae*	225	9.00	Adenovirus + Inf A	7	0.28
RV/EV + *S. pneumoniae*	217	8.68	CoV HKU1 + *S. pneumoniae*	7	0.28
RSV A/B + *S. pneumoniae*	140	5.60	CoV OC43 + PIV 3	7	0.28
Inf A + *H. influenzae*	119	4.76	MPV + SARS-CoV-2	7	0.28
SARS-CoV-2 + *H. influenzae*	109	4.36	PIV 1 + *S. pneumoniae*	7	0.28
RSV A/B + *H. influenzae*	107	4.28	PIV 4 + RSV A/B	7	0.28
Inf A + *S. pneumoniae*	73	2.92	Adenovirus + CoV OC43	6	0.24
SARS-CoV-2 + *S. pneumoniae*	65	2.60	*B. pertussis + H. influenzae*	6	0.24
*Adenovirus + H. influenzae*	63	2.52	BoV + SARS-CoV-2	6	0.24
MPV + *H. influenzae*	61	2.44	CoV NL63 + *H. influenzae*	6	0.24
RV/EV + SARS-CoV-2	54	2.16	CoV OC43 + Inf A	6	0.24
RSV A/B + RV/EV	45	1.80	Inf A + RSV A/B	6	0.24
PIV 3 + RV/EV	44	1.76	Inf B + RV/EV	6	0.24
PIV 3 + *S. pneumoniae*	40	1.60	Adenovirus + BoV	5	0.20
PIV 3 + RSV A/B	39	1.56	CoV 229E + PIV 3	5	0.20
Inf B + *H. influenzae*	35	1.40	CoV 229E + RV/EV	5	0.20
Adenovirus + RV/EV	28	1.12	CoV 229E + *S. pneumoniae*	5	0.20
PIV 3 + *H. influenzae*	28	1.12	CoV NL63 + RV/EV	5	0.20
BoV + RV/EV	27	1.08	CoV OC43 + MPV	5	0.20
CoV OC43 + RV/EV	27	1.08	Parechovirus + *H. influenzae*	5	0.20
Inf B + *S. pneumoniae*	27	1.08	Parechovirus + RV/EV	5	0.20
BoV + *S. pneumoniae*	26	1.04	PIV 2 + *S. pneumoniae*	5	0.20
BoV + RSV A/B	25	1.00	PIV 4 + *S. pneumoniae*	5	0.20
CoV OC43 + SARS-CoV-2	24	0.96	Adenovirus + SARS-CoV-2	4	0.16
MPV + *S. pneumoniae*	24	0.96	BoV + MPV	4	0.16
CoV OC43 + *H. influenzae*	23	0.92	CoV 229E + RSV A/B	4	0.16
CoV OC43 + RSV A/B	20	0.80	CoV HKU1 + SARS-CoV-2	4	0.16
PIV 3 + SARS-CoV-2	20	0.80	SARS-CoV-2 + B. Pertussis	4	0.16
Adenovirus *+ S. pneumoniae*	19	0.76	Adenovirus + Inf B	3	0.12
BoV + PIV 3	17	0.68	Adenovirus + PIV 2	3	0.12
RSV A/B + SARS-CoV-2	17	0.68	*B. pertussis + S. pneumoniae*	3	0.12
BoV + *H. influenzae*	16	0.64	CoV 229E + CoV OC43	3	0.12
BoV + Inf A	13	0.52	CoV 229E + SARS-CoV-2	3	0.12
CoV OC43 + *S. pneumoniae*	13	0.52	CoV HKU1 + RSV A/B	3	0.12
Inf A + RV/EV	13	0.52	CoV OC43 + Inf B	3	0.12
Inf A + SARS-CoV-2	13	0.52	Inf A + PIV 3	3	0.12
MPV + RV/EV	13	0.52	Inf B + RSV A/B	3	0.12
CoV HKU1 + RV/EV	12	0.48	Inf B + SARS-CoV-2	3	0.12
RV/EV + B. Pertussis	12	0.48	Parechovirus + RSV A/B	3	0.12
CoV 229E + *H. influenzae*	10	0.40	PIV 4 + SARS-CoV-2	3	0.12
CoV HKU1 + *H. influenzae*	10	0.40	Adenovirus + PIV 1	2	0.08
PIV 1 + RV/EV	9	0.36	Adenovirus + RSV A/B	2	0.08
PIV 4 + *H. influenzae*	8	0.32	Others	58	2.32

## Data Availability

The data presented in this study are available on request from the corresponding author. The data are not publicly available due to privacy concerns.
